# Impact of BH3-mimetics on Human and Mouse Blood Leukocytes: A Comparative Study

**DOI:** 10.1038/s41598-019-57000-x

**Published:** 2020-01-14

**Authors:** Lionel Rohner, Ramona Reinhart, Joseena Iype, Sofia Bachmann, Thomas Kaufmann, Michaela Fux

**Affiliations:** 1 University Institute of Clinical Chemistry, Inselspital, Bern University Hospital, University of Bern, Bern, Switzerland; 2 Institute of Pharmacology, University of Bern, Bern, Switzerland; 3 Graduate School for Cellular and Biomedical Sciences Bern, University of Bern, Bern, Switzerland

**Keywords:** Cell death and immune response, Cell death and immune response, Immune cell death, Immune cell death

## Abstract

BH3-mimetics are small molecule inhibitors that neutralize the function of anti-apoptotic BCL-2 family members. BH3-mimetics have recently gained a lot of popularity in oncology because of their success in cancer treatment. However, BH3-mimetics might have a broader clinical application. Here, we established an *ex vivo* flow cytometric assay allowing the comparison of the impact of BH3-mimetics (ABT-199, ABT-263, WEHI-539, and S63845) on leukocyte populations of both, healthy human subjects and C57BL/6 J wild type mice. BH3-mimetics were added to freshly drawn blood that was diluted 1/2 in cell medium, and BH3-mimetics-mediated impact on leukocyte count was assessed by flow cytometry. Our results demonstrate that responses towards 1μM of BH3-mimetics can be identical as well as considerably different in leukocytes of humans and mice. For instance, the inhibition of BCL-2 by ABT-199 caused cell death in all types of lymphocytes in mice but was exclusively specific for B cells in humans. Moreover, inhibition of BCL-X_L_ by WEHI-539 affected solely mouse leukocytes while targeting MCL-1 by S63845 resulted in efficient induction of cell death in human neutrophils but not in their mouse counterparts. Our *ex vivo* assay enables initial identification of analogies and differences between human and mouse leukocytes in response towards BH3-mimetics.

## Introduction

Excessive cell death or evasion from apoptosis is associated with the development of autoimmune disorders or cancer^1^. Apoptosis is primarily regulated by B cell lymphoma 2 (BCL-2) family members, which differ based on their ability to promote or to suppress apoptosis by affecting the integrity of the mitochondrial outer membrane (MOM). Anti-apoptotic BCL-2 family members (BCL-2, BCL-X_L_, BCL-W, MCL-1, BFL-1/A1) function by inhibiting their pro-apoptotic counterparts, which, upon their “unleashing“, trigger MOM permeabilization followed by activation of apoptotic caspases, culminating in the ultimate, irreversible cascade towards cellular demise^2,3^. Previous studies have shown that the interplay of pro- and anti-apoptotic BCL-2 members specifically regulates hematopoiesis and survival of leukocytes in blood and bone marrow^4,5^. Importantly, upregulation of certain anti-apoptotic BCL-2 family members, such as BCL-2, B cell lymphoma-extra-large (BCL-X_L_), and myeloid cell leukemia (MCL-1), are also known to support development and survival of cancerous cells as well as evasion from cancer therapy^6^. To overcome such resistance towards apoptosis, small molecule inhibitors called BCL-2 homology domain 3 (BH3) -mimetics have been developed to specifically neutralize anti-apoptotic BCL-2 family members in order to restore normal apoptotic signaling in cancerous cells^7^.

Most studies involving BH3-mimetics have been carried out in cancer cells. However, with some exceptions, the impact of these compounds on key leukocyte populations from healthy individuals is less clear. As the expression of individual anti-apoptotic BCL-2 family members varies substantially between different leukocyte subtypes and their activation statuses, BH3-mimetics may also represent a powerful tool to target specific cell types in diseases other than cancer, for example in immune disorders. In this study, we present a flow cytometry assay that allows tracking changes in the major myeloid and lymphoid cell populations *ex vivo* using whole blood samples. We established an assay valid for both human and mouse derived blood samples, allowing an initial comparison of the response of human and mouse leukocytes towards BH3-mimetics.

## Results and Discussion

In this study, we present an *ex vivo* flow cytometry assay that allows quantitative assessment of human and mouse leukocyte viabilities in whole blood samples in response to BH3-mimetics. We used following BH3-mimetics: ABT-199 (BCL-2 inhibitor), ABT-263 (inhibiting BCL-2, BCL-X_L_, and BCL-W), WEHI-539 (targeting BCL-X_L_), and S63845 (MCL-1 inhibitor).

**Figure 1:**
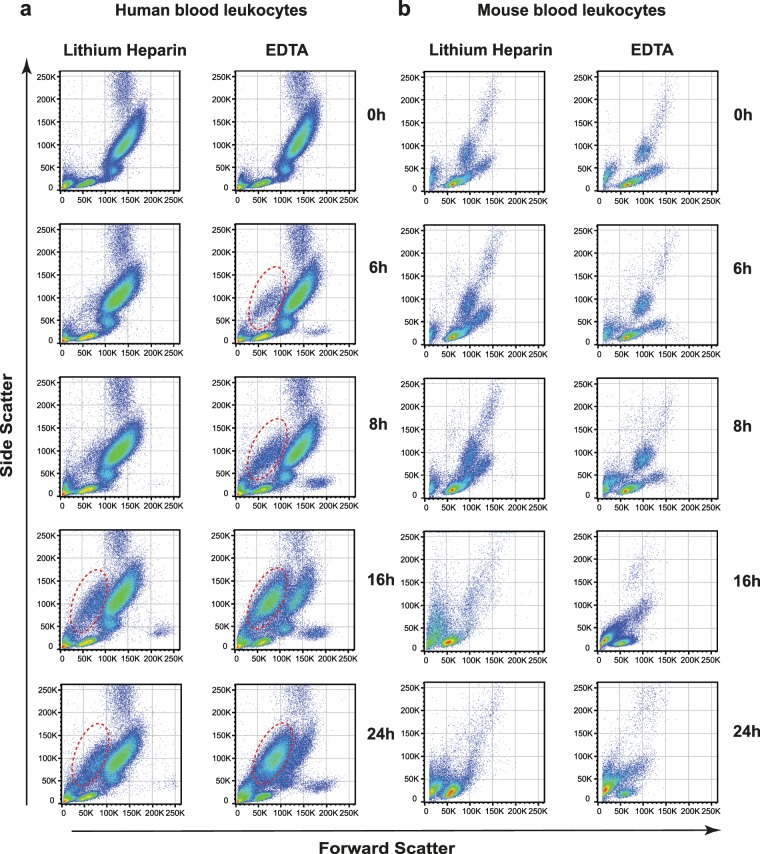
Evaluation of the effect of EDTA and lithium heparin on human and mouse blood *ex vivo* culture conditions. Shown are the light scatter characteristics of human (**a**) and mouse (**b**) blood samples collected in either EDTA or LiHep blood collection tubes, as indicated. Blood samples were diluted 1/2 in cell culturing medium (RPMI+/+) and kept at 37 °C in a 5% CO2 atmosphere for 6, 8, 16 and 24 hours. Fresh blood samples (0 h) were processed immediately after blood sampling.

We aimed to establish an assay to test the effects of BH3-mimetics in human and mouse specimens under non-invasive conditions, which are nevertheless close to physiological conditions. *Ex vivo* conditions using whole blood samples provide such a potential. In order to minimize the impact of pre-analytic factors, we first compared the effect of different anticoagulants on cellular integrity over different time points. We observed that in unfractionated blood cellular integrity is not maintained long enough to test the effect of BH3-mimetics, regardless of the type of anticoagulant used (data not shown). Therefore, and based on our experience with routine testing of patient’s samples, we diluted whole blood samples 1/2 in complete RPMI +/+ cell medium. We observed that in diluted human whole blood samples a portion of the granulocyte population (as identified based on high side scatter) shifted towards the debris section within 6 hours and almost completely lost its initial integrity within 24 hours (Fig. 1a, circle) if ethylenediaminetetraacetic acid (EDTA) was used. However, this effect was negligible in lithium heparin (LiHep) samples. Regardless of the anticoagulant used, human monocytes started to vanish after 16 hours, based on the decrease of the CD14 positive population (see Supplementary Fig. S5). In mouse blood, EDTA and LiHep preparations were comparable, but leukocytes were overall more ephemeral compared to their human counterparts. For the analysis of BH3-mimetics-mediated cell death we intended to use an incubation time that was long enough to detect an effect of BH3-mimetics and that had only a minimal effect on spontaneous cell death. Our previous study has shown that at least 8 hours and ideally, more than 10 hours were necessary to induce significant cell death in human granulocytes^8^. Compared to fresh LiHep samples (0 h), 8 hours for both human and mouse samples and 16 hours for human samples showed the smallest bias of spontaneous cell death. Hence, we decided to expose LiHep blood samples for 8 hours (mouse and human) and 16 hours (only human), respectively.

**Figure 2:**
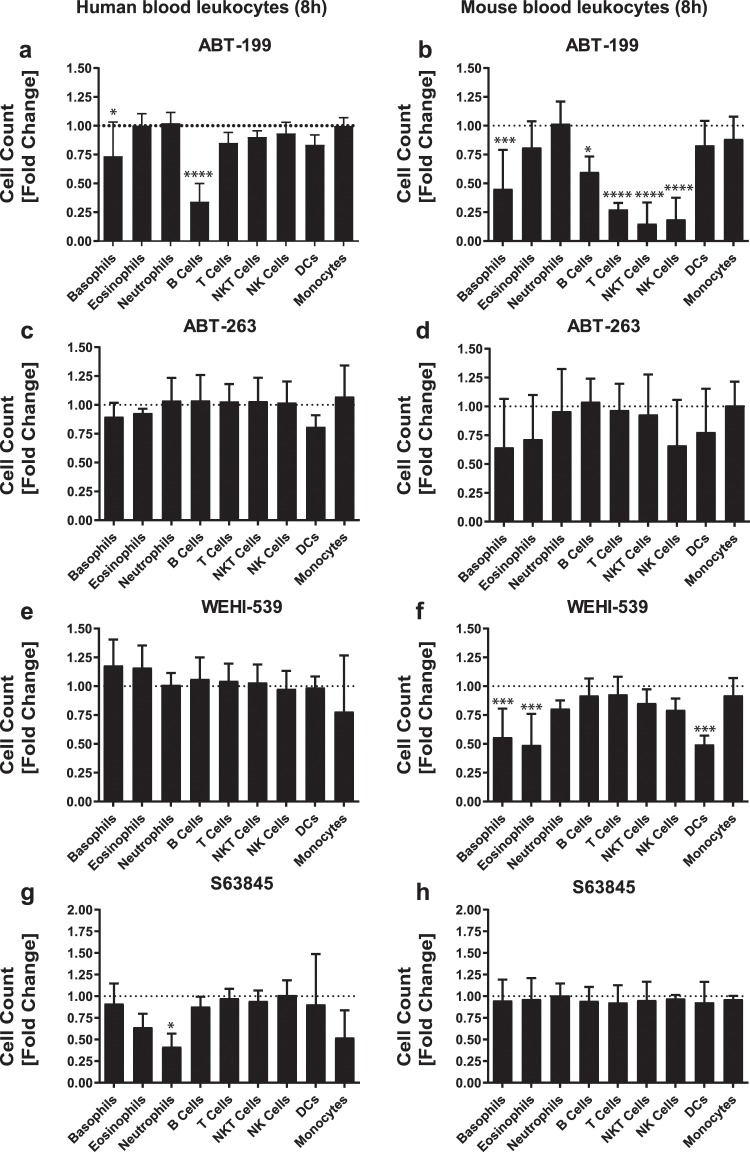
Effect of different BH3-mimetics on the main leukocyte populations in mouse and human whole blood samples. Shown are the effect of ABT-199, ABT-263, WEHI-539, and S63845 on total cell count of major leukocyte subpopulations in human **(a,c,e,g)** and mouse **(b,d,f,h)** blood samples. Mouse and human blood samples were kept under *ex vivo* culture conditions for 8 hours. All BH3-mimetics were used at a final concentration of 1 µM. The cell count of a given cell type in the untreated sample (dotted line) was used as a reference for normalization of the treatment groups. A total of 4 biological replicates are shown for each species. Ordinary one-way ANOVA followed by Dunnett’s posthoc test was used for comparing control and treatment groups for statistical differences. Data are shown as mean (SD). n.s. (not significant); *P < 0.05; **P < 0.01, ***P < 0.001, ****P < 0.0001.

In order to compare the maximum cell inducing effect of BH3-mimetics irrespective of dose, all compounds were used at 1 µM. This concentration was selected based on previous results obtained from *in vitro* differentiated basophils^9^ and on other studies demonstrating that 1 µM does not induce cellular toxicity^10^. We confirmed that specific inhibition of BCL-2 by ABT-199 during 8 hours elicited a significant decrease in human and mouse B cell numbers, whereby the effect was more pronounced in human B cells (compare Fig. 2a,b) and which was further enhanced if human blood samples were exposed to ABT-199 during 16 hours (see Supplementary Fig. S6). In accordance with studies by others^11,12^, our data corroborated that mouse T, NK, and NKT cells are highly susceptible to BCL-2 inhibition (Fig. 2b). In contrast, human T, NK, and NKT cells were only affected if exposed for 16 hours (see Supplementary Fig. S6) but not if incubated for 8 hours (Fig. 2a).

**Figure 3:**
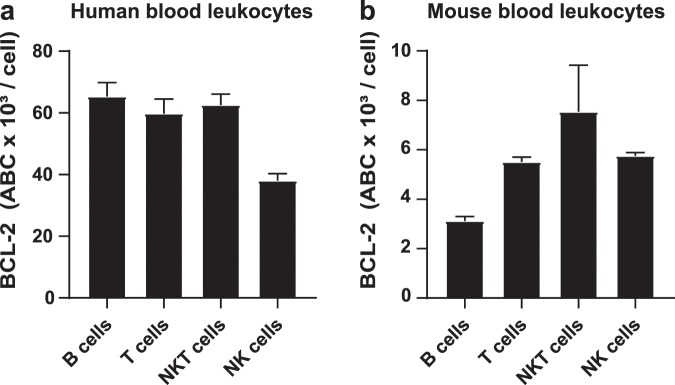
Quantification of absolute BCL-2 molecules per cell in human and mouse lymphocytes. Shown are means ± SEM of absolute numbers of BCL-2 molecules per cell (ABC, antibody binding capacity) in order to allow inter-species comparison of human (**a**) and mouse (**b**) lymphocytes. Mouse and human blood samples were processed immediately after blood sampling. Gating strategy for the identification of lymphocyte subtypes was the same as described in Supplementary Figs. S2 and S4. A total of 3 biological replicates are shown for each species.

In order to evaluate whether the expression levels of the respective targets reflect the sensitivity towards BH3-mimetics, we analyzed the expression of BCL-2 (see Supplementary Fig. S7), upon the inhibition of which prominent differences within and between human and mouse lymphocytes were seen. In order to make a comparison of human and mouse samples possible, we determined absolute BCL-2 molecules per cells by using a commercially available standard consisting of 5 different bead populations. That approach prevents biases due to different instrument settings, compensation and autofluorescence between human and mouse samples. Absolute quantification of BCL-2 molecules per cell revealed that in mouse lymphocytes the higher the level of BCL-2 expression was (NKT > NK = T cells > B cells) (Fig. 3b) the more sensitive they were towards BCL-2 inhibition (NKT = NK = T cells > B cells). Interestingly, human T and NKT cells express up to 10-fold higher levels of BCL-2 than their mouse counterpart (Fig. 3a,b). This species-specific difference in expression of BCL-2 could explain why BCL-2 inhibition was only effective after 16 hours in human cells (see Supplementary Fig. S6), whereas in mouse samples 8 hours were sufficient (Fig. 2b). However, the sensitivity towards ABT-199 cannot in all instances be explained by the expression level of its target. For instance, human B cells express BCL-2 at comparable levels as human T cells of which the latter showed low sensitivity towards ABT-199 treatment. Our findings support the widely accepted view that that the sensitivity towards BH3-mimetics is most probably the sum of the interplay of a network of anti- and pro-apoptotic BCL-2 family members.

We have previously published that ABT-199 potently induces apoptosis in mouse basophils and mast cells^9^ as well as in human basophils, but not in human eosinophils or neutrophils^8^. Our present data confirmed that survival of both human and mouse blood basophils was highly dependent on BCL-2, although with different time dependency. While the numbers of mouse basophils decreased by about 50% in response to ABT-199 treatment at 8 hours already (Fig. 2b) a 50% reduction of human basophils was observed after 16 hours (Fig. 2a and see Supplementary Fig. S6). Nevertheless, this is an interesting result as basophils are associated with inflammatory response in various allergic disorders^13–15^. Therefore, targeting basophils using ABT-199 could be an attractive approach to modulate the outcome of allergic inflammation, considering the fact that after prolonged incubation time of 24 hours, the half-maximal effective concentration of ABT-199 is up to 140-times lower in human basophils than B cells^8,11^. Human neutrophils and eosinophils were not affected by ABT-199 (Fig. 2a), which is in agreement with our former findings^8^.

ABT-263 was a less effective inducer of cell death than ABT-199, affecting significantly human B cells, and basophils but only if exposed longer than 16 hours (Fig. 2c, and see Supplementary Fig. S6). The impact of ABT-263 on leukocyte survival under *ex vivo* conditions at later time points could not be tested in mouse samples, since after 8 hours the cellular integrity seemed not to be guaranteed anymore (Fig. 1b). Moreover, our previously published studies demonstrated that ABT-263 also greatly accelerated spontaneous apoptosis of isolated human eosinophils^8^ and mouse basophils^9^. We assume that a higher concentration of ABT-263 and/or a longer incubation time would be required to achieve a similar response as ABT-199 in human eosinophils and mouse basophils for two reasons. On the one hand, it has been reported that ABT-263 exhibits reduced activity when exposed to plasma proteins^16^ and on the other hand it has a 100 times lower inhibitory constant to BCL-2 than ABT-199 ^17,18^.

In contrast to human leukocytes and mouse T, NK and NKT, which were not responsive to WEHI-539 (Fig. 1e,f), selective inhibition of BCL-X_L_ was effective at killing mouse cells of the myeloid lineage, including basophils, eosinophils and DCs (Fig. 2f). The importance of BCL-X_L_ for the survival of mouse but not human basophils (see Supplementary Fig. S8) is consistent with our previous work using isolated basophils^9^.

The newly developed MCL-1 selective inhibitor S63845 has drawn a lot of attention as MCL-1 emerges as a critical player in conferring chemoresistance, underlined by the frequent amplification of the *MCL-1* gene in a variety of human cancers^19^. While none of the mouse leukocyte populations were affected by 1μM S63845, the number of human neutrophils decreased within 8 hours by over 50% in response to S63845 (Fig. 2g,h), which might be explained by the higher affinity of S63845 towards human MCL-1 compared to mouse MCL-1^20^. Moreover, a recently published study demonstrated that up to 10μM of S63845 are needed to induce significant cell death in short-termed murine AML cell lines^21^. Hence, we assume that higher concentration of S63845 is needed to efficiently trigger cell death in mouse leukocytes. The susceptibility of human neutrophils to S63845 was anticipated as survival of this cell types has been linked to MCL-1^22^. Moreover, and in line with a former study showing that peripheral blood B cell survival correlates with MCL-1 levels^23^, our data - albeit not statistically significant – demonstrate that human B cell numbers fell by over 25% within 16 hours upon MCL-1 antagonism (see Supplementary Fig. S6).

Our *ex vivo* flow cytometry approach showed that inhibition of essential anti-apoptotic proteins differently impacts the absolute cell count of major leukocyte populations circulating in venous blood of humans and mice, whereby ABT-199 and WEHI-539 treatment groups exhibited the statistically most significant differences after 8 hours incubation (see Supplementary Fig. S8). Our data underline that the effect of BH3-mimetics differ between human and mouse leukocytes. It remains to be tested whether strain-to-strain differences exist in the mouse; however, the extrapolation from mouse-derived data to the human needs to be critically examined.

## Materials and Methods

### Blood withdrawal

This study has been approved by the Ethics Committee of the canton of Bern (Kantonale Ethikkomission Bern; Nr. 2016-01571) and was conducted according to the criteria set by the declaration of Helsinki. Human blood was collected from voluntary healthy donors after receiving informed consent, using S-Monovettes containing either EDTA or LiHep as anticoagulant (Sarstedt AG and Co. KG, Nümbrecht, Germany). C57BL/6 mice were maintained under pathogen-free conditions in individually ventilated cages (IVC). All animal experiments were approved by the animal experimentation review board of the canton of Bern (BE138/16) and performed in compliance with the humane care and use of laboratory animals and the Swiss animal protection act. Mouse blood was terminally isolated from C57BL/6 J wild type mice after isoflurane-induced anesthesia (Forene®, AbbVie AG, Baar, Switzerland) by periorbital sinus puncture and collected through Na-heparinized Micro Hematocrit-tubes (Henry Schein, Melville, NY, US) into EDTA or LiHep-coated Microvettes (Sarstedt AG and Co).

### Reagents

Complete medium RPMI +/+ consists of RPMI 1640 supplemented with 10% FCS, Penicillin, Streptomycin (1/100 each) and 10 mM HEPES. RPMI 1640 Medium, Penicillin and Streptomycin were obtained from Biochrom (Berlin, DE). Fetal calf serum (FCS, Sera Pro, ultra-low endotoxin) and HEPES were purchased from Pan Biotech (Aidenbach, DE) and Gibco®, Fischer Scientific, respectively. ABT-199 (Venetoclax) was obtained by BioVision (Milpitas, CA, US), ABT-263 (Navitoclax) by Selleck Chemicals (Houston, TX, US), WEHI-539 hydrochloride was purchased from Hycultec (Beutelsbach, DE), S63845 from ApexBio (Houston, TX, US). A stock solution (10 mM) of every type of used BH3-mimetics was prepared in DMSO.

### Establishment of *ex vivo* conditions

To evaluate the impact of different types of anti-coagulants, EDTA and LiHep blood samples of humans and mice were diluted 1/2 in complete RPMI +/+ medium in order to support cell integrity. Blood samples were distributed in 96-well U-bottom tissue-culture plates (BD Falcone, Franklin Lakes, NJ) (200 μl/well). Samples were incubated at 37 °C, 5% CO_2_ for 6 h, 8 h, 16 h and 24 h without any stimuli. Afterward incubation, red blood cells of EDTA and LiHep blood samples were lysed using BD Lysing Solution (BD Biosciences) for 15 minutes at room temperature. Thereafter, samples were washed and resuspended in 600 μl Staining Buffer, which was composed of 1x PBS with 2% heat-inactivated FCS and 0.05% sodium azide (Merck Millipore, Zug, Switzerland). Fresh EDTA and LiHep blood samples that were immediately after blood withdrawal processed for flow cytometry analysis (0 h) were used as references. Data were acquired using a BD FACS Canto II flow cytometer (BD Bioscience).

### Stimulation of LiHep blood samples with BH3-mimetics

In order to test the effect of BH3-mimetics on different human and mouse leukocyte populations, LiHep blood samples were diluted and distributed in 96-well plates as mentioned above whereby mouse blood was pooled from 3–4 C57BL/6 J inbred wild type mice to collect enough blood for all treatment groups. Samples were exposed to medium only or to different BH3-mimetic compounds (ABT-199, ABT-263, WEHI-539, or S63845) all used at 1 µM, which was prepared by diluting the 10 mM stock solution in RPMI +/+ medium. After 8- and 16-hours incubation, mouse and human blood samples, respectively were processed for flow cytometric analysis.

### Flow cytometric assessment of the response of blood leukocytes towards BH3-mimetics

After exposing to BH3-mimetics, LiHep blood samples were stained with Yellow-Green Live Cell Caspase Probe (BD Biosciences, San Jose, CA, US) according to manufacturer instructions. Cells were washed with 1x PBS followed by staining with fluorochrome-labeled antibodies (listed below) for 15 minutes at room temperature. Afterward, red blood cells were lysed as mentioned above. Thereafter, samples were washed and resuspended in 600 μl Staining Buffer. CountBright^TM^ absolute counting beads (Invitrogen, Basel, Switzerland) were added to the lysed blood samples shortly before analysis using a BD FACS Canto II flow cytometer (BD Bioscience). The following panels were used to identify blood leukocytes:

**Human panel 1** (100 μl human blood) was used to detect basophils, eosinophils, and DCs using the following antibodies: anti-human Lin1 FITC (clones 3G8, L27, MϕP9, NCAM16.2, SJ25C1, SK7) (BD Biosciences), anti-human HLA-DR BV421 (clone L243), anti-human CD11c BV510 (clone 3.9), anti-human Siglec-8 PerCP/Cy5.5 (clone 7C9), anti-human CD193 Alexa 647 (clone 5E8), anti-human CD123 PE/Cy7 (clone 6H6) all purchased from BioLegend (San Diego, CA, US). **Human panel 2** (50 μl human blood) was used to detect major human lymphocyte populations (T cells, B cells, NK cells, and NKT cells) and neutrophils. Following antibodies were used: anti-human CD19 BV421 (clone HIB19), anti-human CD16 BV510 (clone 3G8), anti-human CD14 APC (clone HCD14), anti-human CD56 BV510 (clone 5.1H11), anti-human CD66b PE/Cy7 (clone G10F5) all from BioLegend, and anti-human CD3 FITC (clone SK7) and anti-human CD45 PerCP (clone 2D1) (both from BD Biosciences, Allschwil, CH). The choice of the surface antigens for the identification of the different types of human blood leukocytes was based on standardized gating strategies from our routine diagnostic cytomics facility at the Centre of Laboratory Medicine at the Inselspital Bern (see Supplementary Figs. S1 and S2). **Mouse panel 1** (200 μl mouse blood) was used to identify basophils, eosinophils, neutrophils and DCs using following antibodies: anti-mouse IgE FITC (clone RME-1), anti-mouse CD11c BV510 (clone N418), anti-mouse CD117/c-Kit PE/Cy7 (clone ACK2), anti-mouse Ly-6G PerCP (clone 1A8), anti-mouse CD19 APC/Cy7 (clone 6D5), anti-mouse NK1.1 APC/Cy7 (clone PK136), anti-mouse CD3 APC/Cy7 (clone 17A2), anti-mouse CD49b APC (clone DX5) (all from BioLegend), and anti-mouse CD170/Siglec F Super Bright 436 (clone 1RNM44N) (Invitrogen). **Mouse panel 2** (100 μl mouse blood) was used to detect B cells, NK cells, NKT cells and T cells and consisted of following antibodies: anti-mouse CD19 BV421 (clone B4), anti-mouse CD45 PerCP (clone 30-F11), anti-mouse CD3 APC/Cy 7 (clone 17A2), anti-mouse NK1.1 BV510 (clone PK136) anti-mouse CD14 APC (clone Sa14-2) all purchased from BioLegend. Gating strategies used to define mouse blood leukocytes were adapted to a great extent from previous studies (see Supplementary Figs. S3 and S4)^19^.

### Detection of intracellular BCL-2 by flow cytometry

Intracellular BCL-2 expression was assessed in fresh EDTA blood samples of humans and mice. Surface staining of unfractionated blood samples was performed as mentioned above using the same antibody panels except that Yellow-Green Live Cell Caspase Probe was omitted. After lysis of red blood cells, samples were washed, and 1 ml of BD Lysing Solution (BD Biosciences) was added in order to permeabilize the cells. Samples were incubated at 37 °C for 30 minutes. Cells were subsequently washed and stained with anti-BCL-2 PE (clone 124 for anti-human and clone 10C4 for anti-mouse) for 30 minutes at room temperature. Thereafter, samples were washed and resuspended in 600 μl Staining Buffer. In order to compare BCL-2 expression between human and mouse samples, absolute BCL-2 molecules per cell were determined using Quantum Simply Cellular Beads (Bangs Laboratories) according to manufacturer’s instructions. Briefly, the BCL-2 molecules per cell were interpolated using a standard consisting of 5 bead populations with increasing capacity to bind anti-BCL-2 PE through increasing levels of Fc-specific capture antibody. Subsequently, the signal of the PE FMO sample was subtracted from the stained samples. The results are shown as antibody binding capacity (ABC) per cell.

### Data analysis

Raw flow cytometry data were analyzed by Flow Jo 10.5.0 (Tree Star Inc., Ashland, OR, US). In order to determine total cell count, raw flow cytometry data were cleaned from CountBright^TM^ absolute counting beads (based on high fluorescence and scatter homogeneity) and doublets (based on area to height ratio of the forward scatter). Anucleated and non-viable cells were removed from further analysis by setting a threshold on low forward scatter values and events positive for Yellow-Green Live Cell Caspase Probe. Total cell count for each sample was calculated using CountBright^TM^ absolute counting beads according to the manufacturer’s instructions.

### Statistical analysis

The alpha level was set to 0.05. Data are expressed as mean and standard deviation (SD) or standard error of mean (SEM) as indicated. Cell count was normalized relative to untreated control samples in order to compensate for inter-donor variability. Statistical analysis of group comparison was performed using ordinary one-way ANOVA followed by Bonferroni’s or Dunnett’s post hoc test as indicated post hoc test using Prism 7 software (GraphPad, La Jolla, CA, US).

## Supplementary information

Supplementary Information.

## Data Availability

For original data, please contact michaela.fux@insel.ch.
