# Supplementary material for: Impact of BH3-mimetics on Human and Mouse Blood Leukocytes: A Comparative Study
Source: Sci Rep. 2020 Jan 14;10:222. doi: 10.1038/s41598-019-57000-x (PMC6959258; doi:10.1038/s41598-019-57000-x)
Supplement: Supplementary file 1 — Supplementary Information. [file 41598_2019_57000_MOESM1_ESM.pdf]

Impact of BH3-mimetics on Human and Mouse Blood Leukocytes: A Comparative Study

Lionel Rohner[1,3†], Ramona Reinhart[2,3†], Joseena Iype[1], Sofia Bachmann[1], Thomas Kaufmann[2], and Michaela Fux[1*]

[1]University Institute of Clinical Chemistry, Inselspital, Bern University Hospital, University of Bern, Switzerland

[2]Institute of Pharmacology, University of Bern, Bern, Switzerland

[3]Graduate School for Cellular and Biomedical Sciences Bern, University of Bern, Switzerland

[†]the first two *authors* are *co-first authors*

[*]corresponding author: Michaela Fux, michaela.fux@insel.ch

Supplementary Figure Legends

Supplementary Figure 1. Gating strategy for the identification of human basophils, eosinophils, and DCs. (a) In a first step CountBright™ Absolute Counting Beads were excluded from further analysis based on high fluorescence in the PerCP channel. This was followed by the exclusion of debris, doublets, caspase positive cells, and Lin1+ cells to allow better cell-specific gating. **(b)** Basophils were identified based on low SSC, dual expression of CD123 and CD193 and absence of HLA-DR and CD11c. **(c)** Eosinophils were defined as SSC high, Siglec 8+, CD193+, HLA-DR- and CD11c- cells. **(d)** The DC population was summarized as pDC and mDCs due to low counts, whereby the former were identified as HLA-DR+, CD123+ and CD11c+ and the latter as HLA-DR+, CD123-, CD11c+. (**b-d)** Backgating of the leukocyte population (red dots) is depicted on top of all the cells that are negative for the Caspase Probe (blue).

Supplementary Figure 2. Gating strategy for the identification of human lymphocytes (T-, B-, NK, and NKT Cells), monocytes and neutrophils. (a) To minimize background and fluorescence artifacts CountBright™ Absolute Counting Beads, doublets, debris, caspase probe+, and CD45- were excluded from further analysis. **(b)** T cells were identified as CD3+, CD19-, CD16- and CD56- (**c**), NKT cells were defined as CD3+, CD19-, CD16/CD56+ cells. **(d)** NK cells were defined as SSC low, CD16/56+, CD66b-, CD14-, CD3- and CD19- events. **(e)** Neutrophils were identified based on high SSC, expression of CD66b and lack of CD3 and CD19. **(f)** B cells were identified as CD19+, CD16/CD56- and CD66b-. (**g**) Monocytes were identified as CD3-, CD14+, CD19- (**b-g)** Backgating of the leukocyte population (red dots) is depicted on top of all the cells that are negative for the Caspase Probe (blue).

Supplementary Figure 3. Gating strategy for the identification of mouse granulocytes (basophils, eosinophils, and neutrophils), and DCs. (a) CountBright™ Absolute Counting Beads, doublets, debris, caspase probe+ and cells positive for lymphocyte markers (CD3, CD19 and NK1.1) were excluded from further analysis. **(b)** Basophils were identified based on expression of CD49b+, IgE+ and absence of CD117, CD11c, Siglec F and Ly6G. **(c)** Eosinophil population was defined as SSC high, Siglec F+, IgE-, Ly6G-, CD11c- and CD117-. **(d)** Neutrophils were identified based on intermediate SSC and high Ly6G expression and lacking surface expression of Siglec F, IgE, and CD117. **(e)** SSC low, CD11c+, CD117-, CD49b-, Ly6G-, and Siglec F- population was defined as dendritic cells (DC). (**b-e)** Backgating of all leukocytes (red dots) is depicted on top of all the cells that are negative for the Caspase Probe (blue).

Supplementary Figure 4. Gating strategy for the identification of major mouse lymphocytes populations (T-, B-, NK, and NKT Cells) and monocytes. (a) CountBright™ Absolute Counting Beads, doublets, debris, caspase probe+, and CD45- events were excluded for all further cell-specific gating strategies. **(b)** T cells were identified based on low SSC, CD3+, CD19- and NK1.1-. **(c)** Except for being positive for NK1.1, NKT cells were identified based on the same strategy as T cells. **(d)** NK cells were defined as CD3-, NK1.1+, and CD19-. **(e)** B cells were defined as CD3-, CD19+, and NK1.1-. **(f)** Monocytes were defined as CD3-, CD14+ CD19-, and NK1.1- (**b-e)** Backgating of the leukocytes (red dots) is depicted on top of all the cells that are negative for the Caspase Probe (blue).

Supplementary Figure 5. Human CD14 positive monocytes disappear spontaneously without the addition of BH3-mimetics within 16 hours. (a) Monocytes were defined as cells with intermediate SSC and high CD14 surface expression. Depicted are 4 biological replicates right after blood donation and after 16 hours.

Supplementary Figure 6. Effect of different BH3-mimetics on human leukocyte populations after 16 hours incubation. Shown are the effect of ABT-199, ABT-263, WEHI-539, and S63845 on total cell count of major leukocyte subpopulations in human blood samples (**a-d**). Human blood samples were kept under *ex vivo* culture conditions for 16h. All BH3-mimetics were used at a final concentration of 1 μM. The cell count of a given cell type in the untreated sample (dotted line) was used as a reference for normalization of the treatment groups. A total of 4 biological replicates are shown. Ordinary one-way ANOVA followed by Dunnett's posthoc test was used for comparing control and treatment groups for statistical differences. Data are shown as mean (SD). n.s. (not significant); *P < 0.05; **P < 0.01, ***P < 0.001, ****P < 0.0001.

Supplementary Figure 7. BCL-2 expression in human and mosue lymphocytes. Shown are representative histograms of human (**a**) and mouse (**b**) lymphocytes. BCL-2 expression (gray area) within each lymphocyte subtype was compared to Fluorescence Minus One (FMO) control of anti-BCL-2 PE (line).

Supplementary Figure 8. Heat map representing the main differences of the inhibition of BCL-2, BCL-$X_{L,}$ and MCL-1 between mouse and human blood leukocytes after 8 hours incubation. (a) The magnitude of the effect of all tested BH3-mimetics was defined as the reciprocal value of the normalized cell count difference between treated and untreated samples. The magnitude of the reduction of human leukocytes was subtracted from its mouse

equivalent and was used to visualize the difference between both species. Resulting positive and negative values near 0 (black) correspond to comparable responses to a given stimulus between both species. Cell types that are affected to a greater extent in mouse or human blood are represented in green and red, respectively. Color intensity reflects the magnitude of the effect on cell count. **(b)** The P-values resulting from the ordinary one-way ANOVA group comparison between human and mouse leukocytes are presented in tabular form. Multiple comparisons were corrected with the Bonferroni's posthoc test. P-values below the α significance level of 0.05 are underlined.

Supplementary Figure 1

Human Panel 1

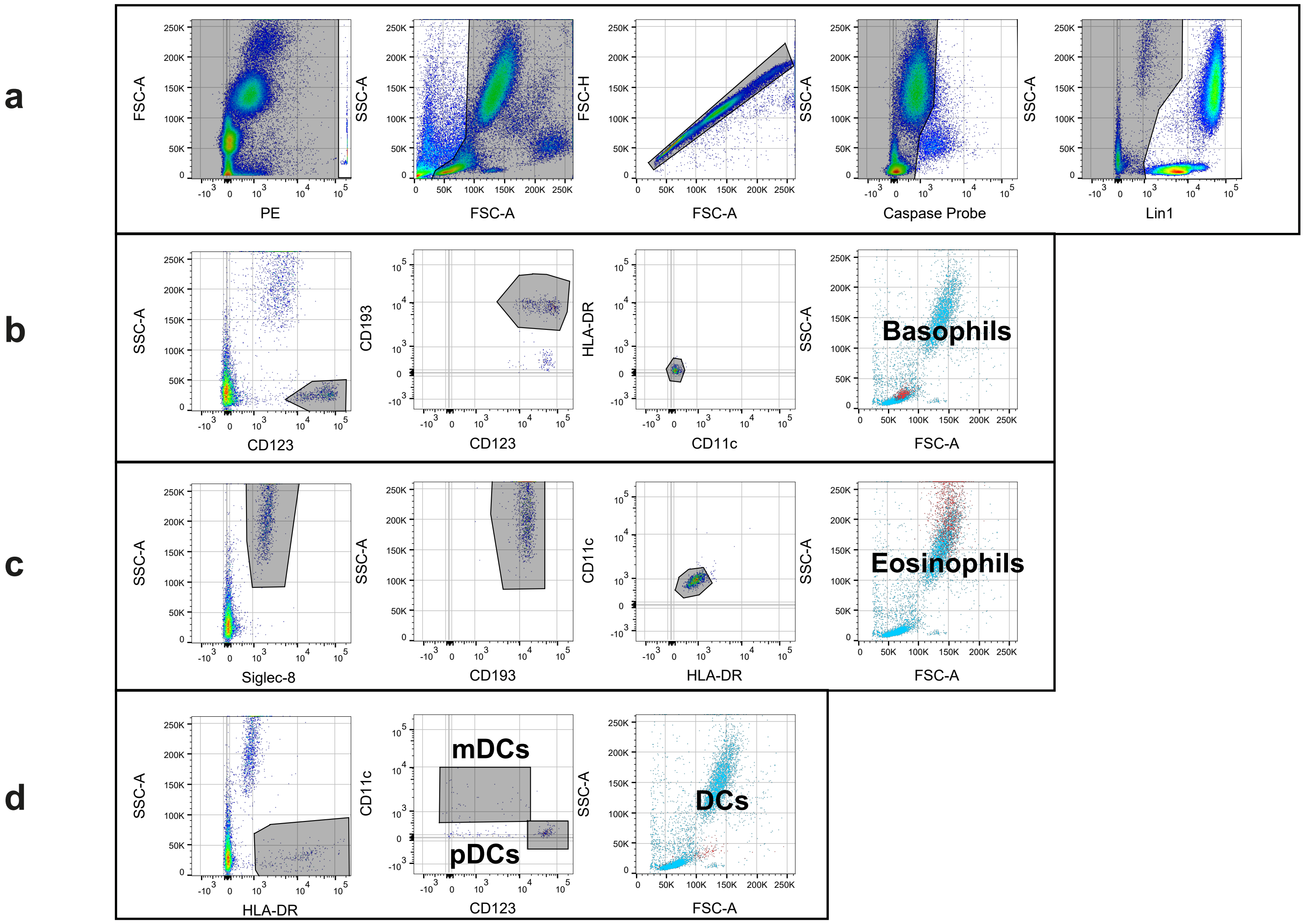

Supplementary Figure 2

Human Panel 2

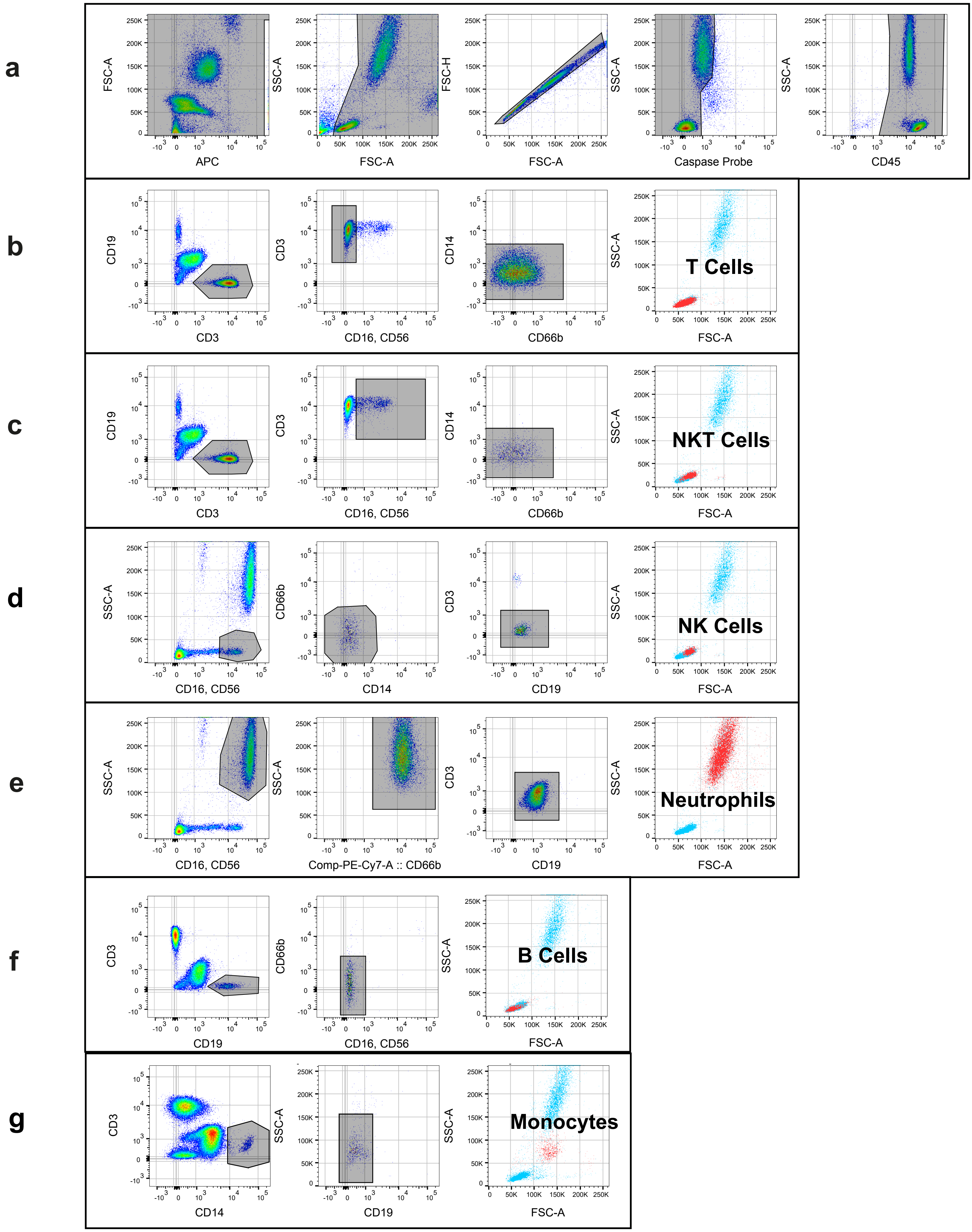

Supplementary Figure 3

Mouse Panel 1

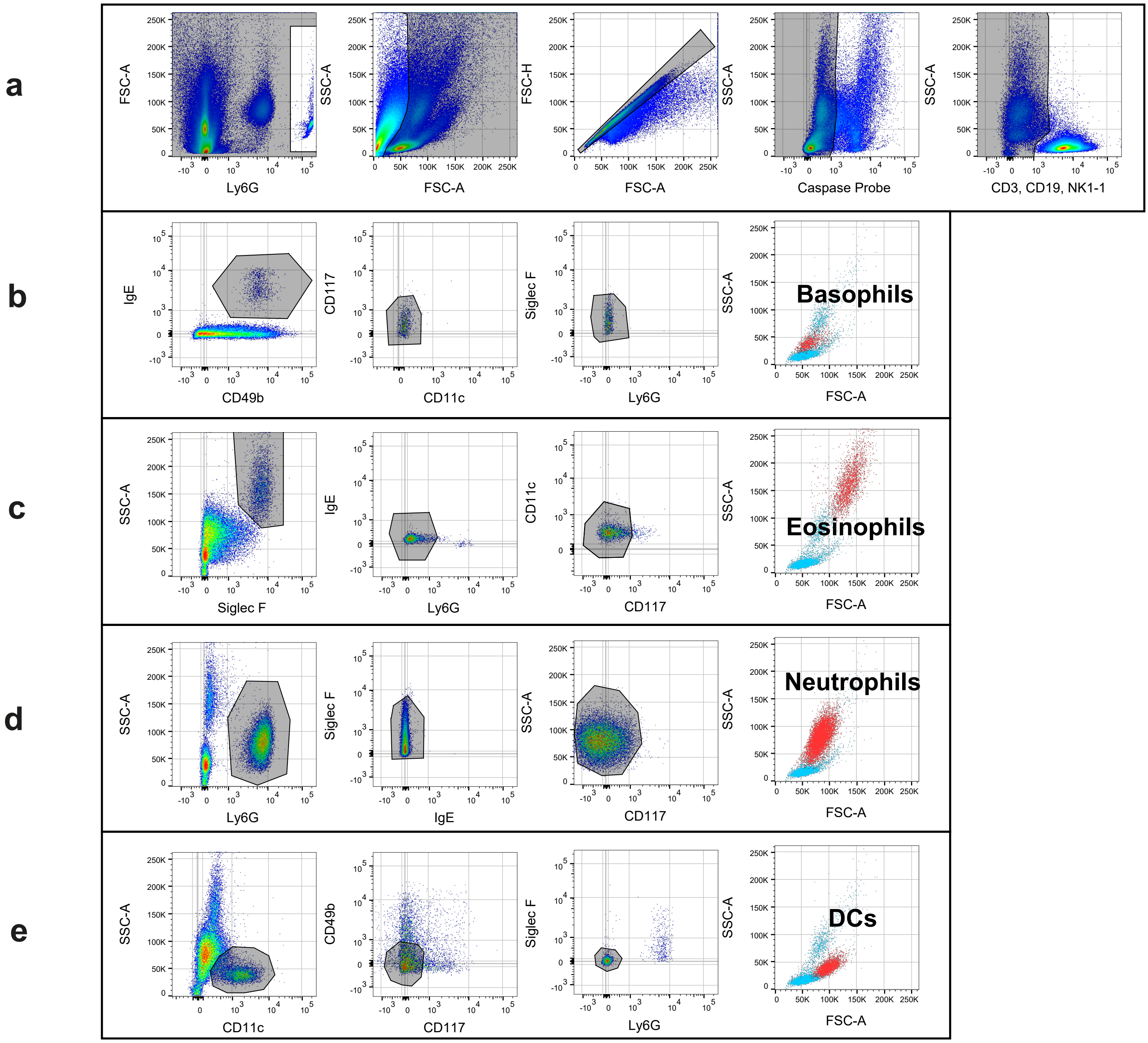

Supplementary Figure 4

Mouse Panel 2

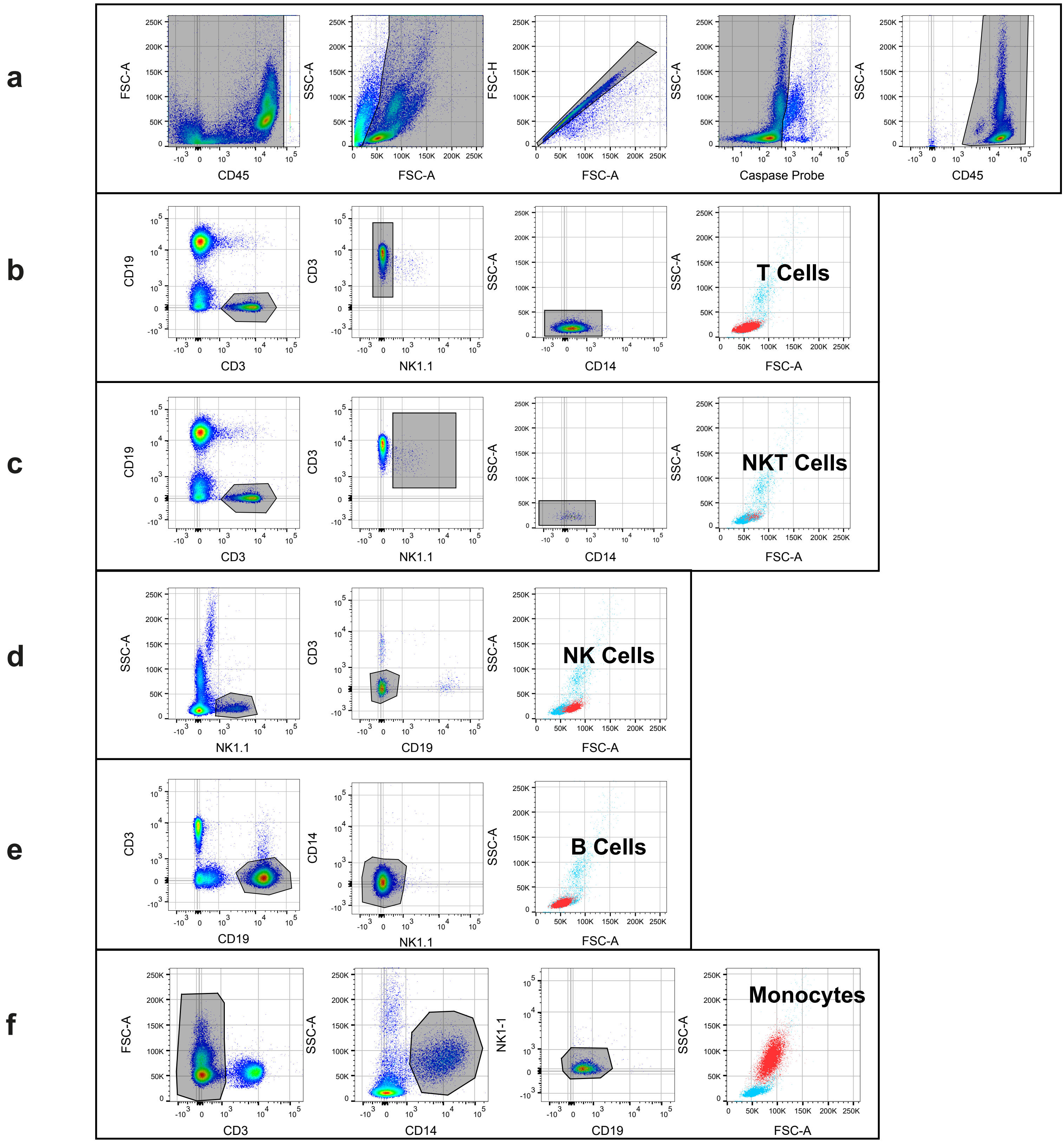

Supplementary Figure 5

Untreated Human Blood Leukocytes

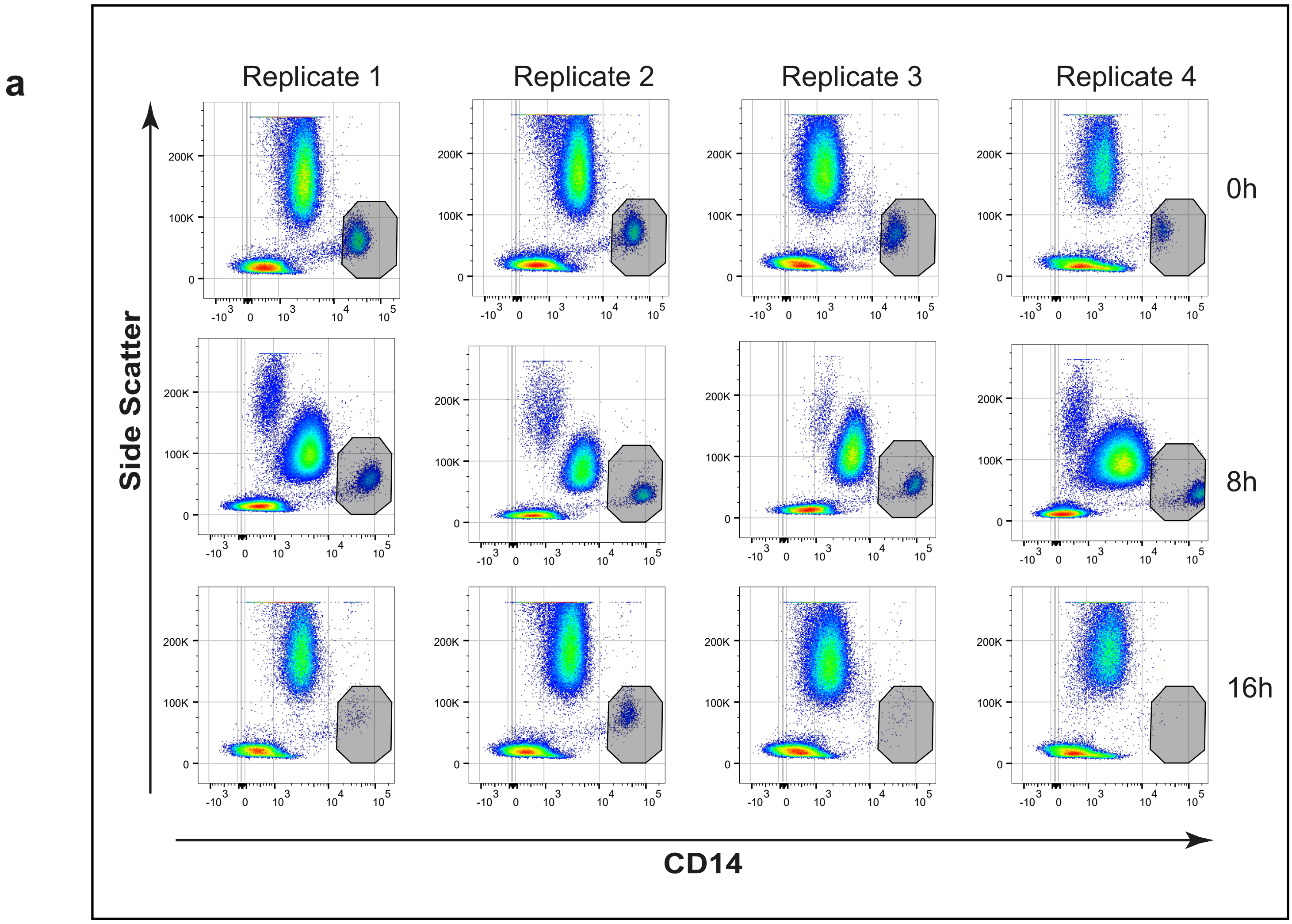

Supplementary Figure 6

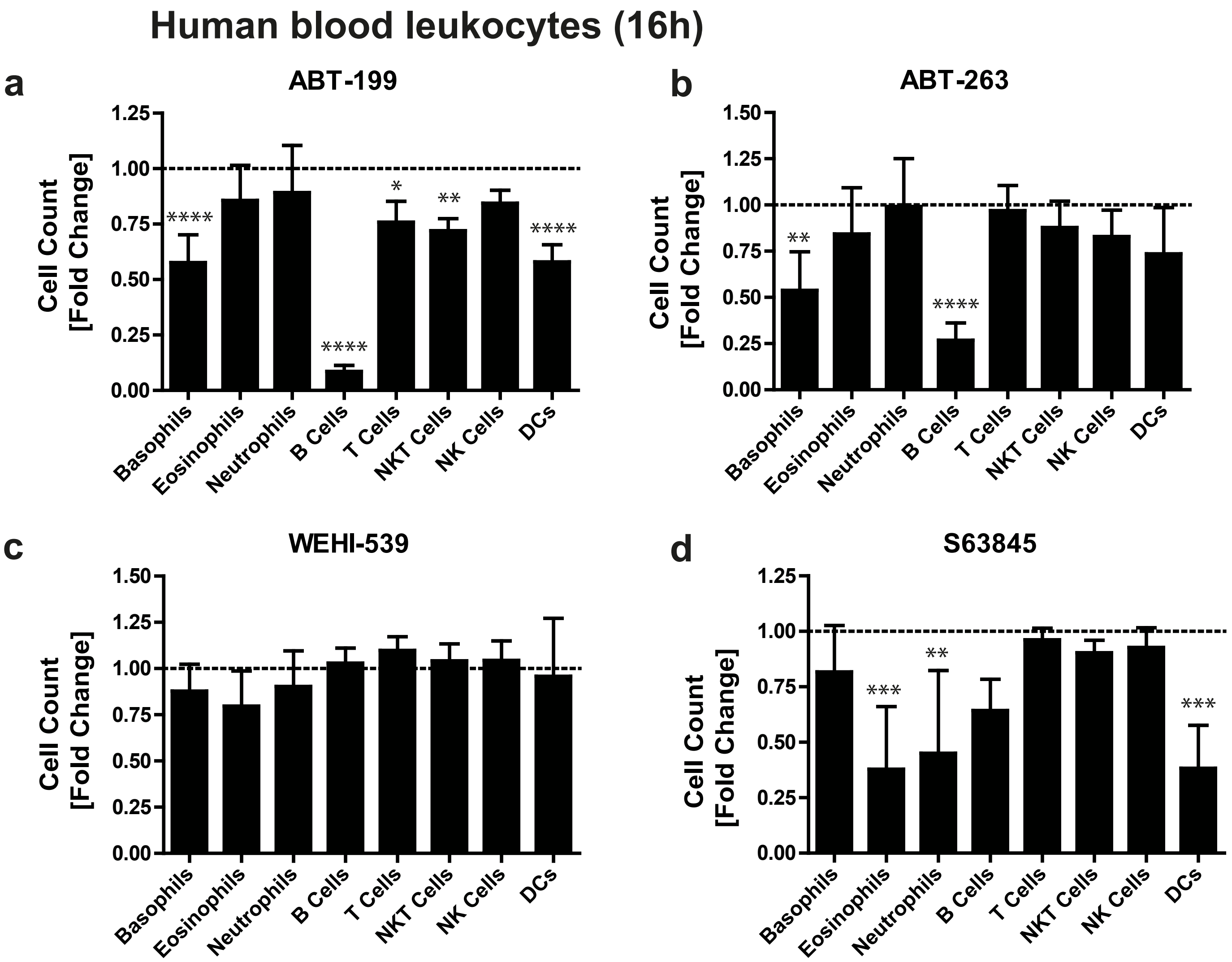

Supplementary Figure 7

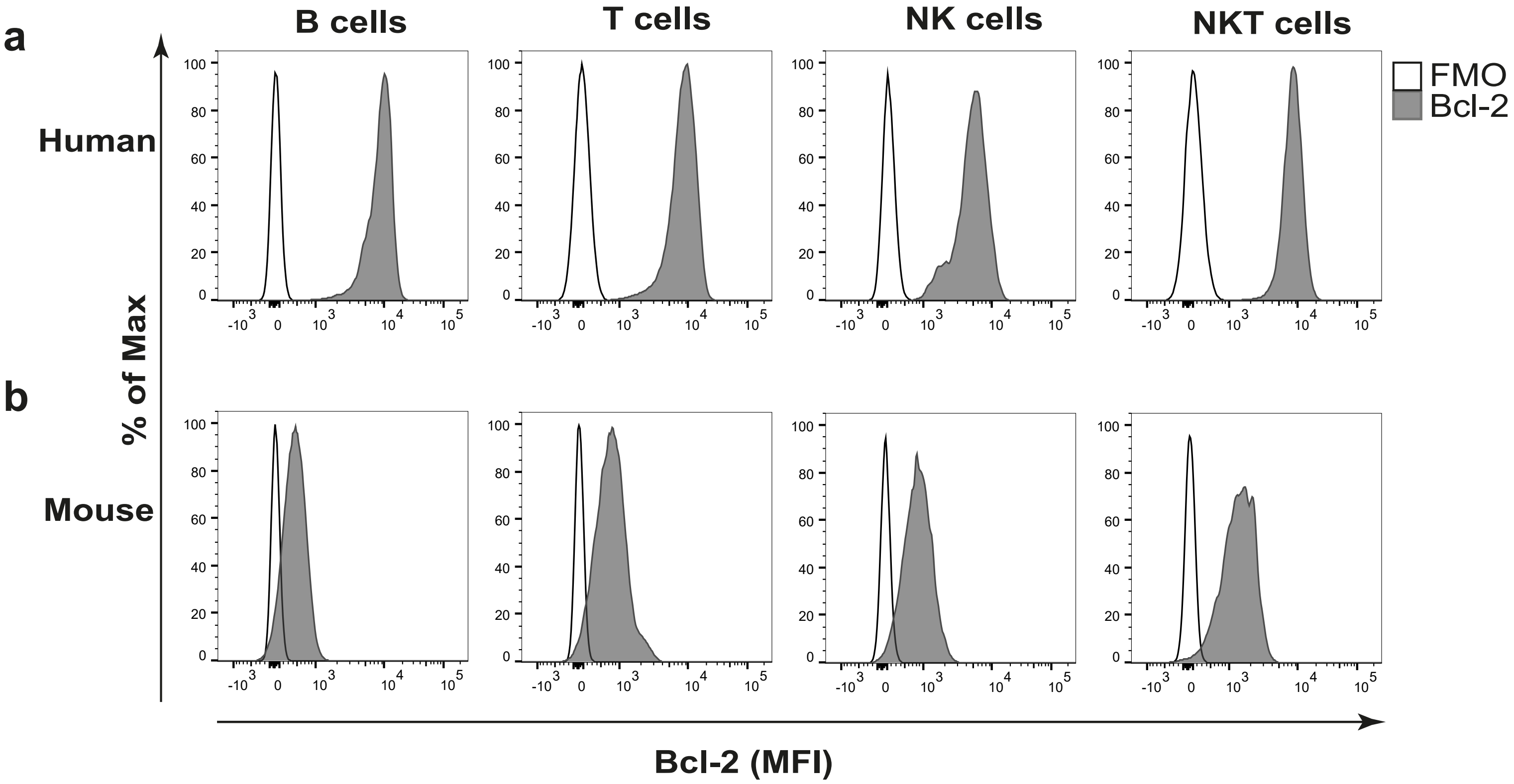

Supplementary Figure 8

a

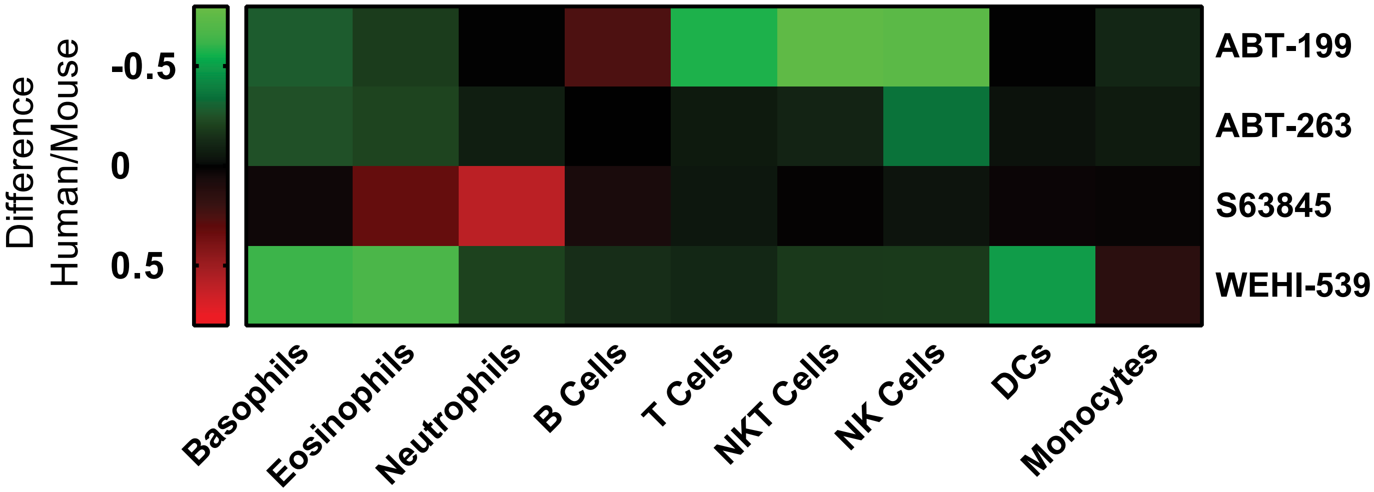

b

One-way ANOVA (Bonferroni)	Adjusted P-values ABT-199	ABT-263	S63845	WEHI-539
Basophils (human) vs. Basophils (mouse)	0.1835	>0.9999	>0.9999	<u>0.0002</u>
Eosinophils (human) vs. Eosinophils (mouse)	>0.9999	>0.9999	0.6468	<u><0.0001</u>
Neutrophils (human) vs. Neutrophils (mouse)	>0.9999	>0.9999	0.057	>0.9999
B Cells (human) vs. B Cells (mouse)	0.4274	>0.9999	>0.9999	>0.9999
T Cells (human) vs. T Cells (mouse)	<u>0.0002</u>	>0.9999	>0.9999	>0.9999
NKT Cells (human) vs. NKT Cells (mouse)	<u><0.0001</u>	>0.9999	>0.9999	>0.9999
NK Cells (human) vs. NK Cells (mouse)	<u><0.0001</u>	0.6318	>0.9999	>0.9999
DCs (human) vs. DCs (mouse)	>0.9999	>0.9999	>0.9999	<u>0.0078</u>
Monocytes (human) vs. Monocytes (mouse)	>0.9999	>0.9999	>0.9999	>0.9999
